# Compromised Motor Dexterity Confounds Processing Speed Task Outcomes in Stroke Patients

**DOI:** 10.3389/fneur.2017.00484

**Published:** 2017-09-21

**Authors:** Essie Low, Sheila Gillard Crewther, Ben Ong, Diana Perre, Tissa Wijeratne

**Affiliations:** ^1^Department of Neurology, Sunshine Hospital, Western Health, Melbourne, VIC, Australia; ^2^Department of Psychology and Counselling, School of Psychology and Public Health, La Trobe University, Bundoora, VIC, Australia; ^3^Department of Psychology, Sunshine Hospital, Western Health, Melbourne, VIC, Australia; ^4^Department of Medicine, Melbourne Medical School, University of Melbourne, Western Health Sunshine Hospital, St Albans, VIC, Australia; ^5^Department of Medicine, University of Rajarata, Anuradhapura, Sri Lanka

**Keywords:** processing speed, motor speed, cognitive speed, stroke, transient ischemic attack, motor impairment

## Abstract

Most conventional measures of information processing speed require motor responses to facilitate performance. However, although not often addressed clinically, motor impairment, whether due to age or acquired brain injury, would be expected to confound the outcome measure of such tasks. The current study recruited 29 patients (20 stroke and 9 transient ischemic attack) with documented reduction in dexterity of the dominant hand, and 29 controls, to investigate the extent to which 3 commonly used processing speed measures with varying motor demands (a Visuo-Motor Reaction Time task, and the Wechsler Adult Intelligence Scale-IV Symbol Search and Coding subtests) may be measuring motor-related speed more so than cognitive speed. Analyses include correlations between indices of cognitive and motor speed obtained from two other tasks (Inspection Time and Pegboard task, respectively) with the three speed measures, followed by hierarchical regressions to determine the relative contribution of cognitive and motor speed indices toward task performance. Results revealed that speed outcomes on tasks with relatively high motor demands, such as Coding, were largely reflecting motor speed in individuals with reduced dominant hand dexterity. Thus, findings indicate the importance of employing measures with minimal motor requirements, especially when the assessment of speed is aimed at understanding cognitive rather than physical function.

## Introduction

Information processing speed is defined as the efficiency or rate of processing information, and is known to be intrinsically related to an individual’s cognitive ability (1). In addition, information processing speed is one of many cognitive domains often assessed by clinicians, as it is almost always compromised following a neurological insult or injury. In particular, reduced speed has been documented in individuals who sustain a traumatic brain injury (2, 3), in patients with multiple sclerosis (4, 5), as well as in other forms of acquired brain injury, including stroke (6, 7).

While many statistical studies have shown that information processing speed can be divided into multifactorial or subdomains of speed factors (8–12), there is no agreed consensus or gold standard for what these speed factors should be. Chiaravalloti et al. (10), for example, have suggested that speed domains should include simple and complex processing speed, while Roberts and Stankov (12) emphasized the importance of perceptual, inductive reasoning, decision and movement, and visual scanning speed. More recently, Knowles et al. (13) have argued for psychomotor, sequencing and shifting, and verbal fluency speed. By comparison, we propose that at the most basic level, rate of information processing should include at least two major domains, the first being a speed domain for any initial *non-motor or cognitive* activity (e.g., perceptual speed for attentional activation, auditory learning or auditory processing speed), and the second for the subsequent *motor or physical* activity that follows (e.g., psychomotor speed, reaction time, and eye movement or saccadic latency).

Currently, clinical measures of speed such as Coding and Symbol Search from the Wechsler Adult Intelligence Scale (WAIS) (14) utilize a conventional paper-and-pencil approach that requires motor responses beyond many other cognitive elements. For example, while the Coding subtest of the WAIS (4th edition) requires selective attention for stimulus recognition, working memory to keep track of the number-symbol code, and decision making to correctly match numbers to symbols, the task additionally requires efficient oculomotor function for visual scanning and fine hand motor control to transcribe symbols (15). Indeed, there has been a growing body of evidence to suggest that psychomotor speed is a major determinant of performance on these speeded measures (16–21). For example hierarchical regressions conducted by Crowe et al. (11) have revealed motor execution abilities to be the strongest predictor of performance on Coding from the WAIS-III, Symbol Search, and Symbol Digit Modalities Test. Furthermore, in a recent study by Ebaid et al. (17), the authors demonstrated significant age-group differences between a young adult and older adult cohort on tasks of Symbol Search and Coding, whilst this significant difference was absent on a non-motor processing speed task known as Inspection Time.

The above findings have important implications for clinical assessments, especially for patients with neurological disorders where both cognitive and motor abilities are concurrently affected. In the context of an ischemic stroke, patients are often left with residual debilitating physical deficits in addition to cognitive deficits, making it likely for speed outcomes on paper-and-pencil measures to predominantly capture the motor speed compromise in these individuals. If a task was indeed more largely measuring motor speed deficits in motor impaired individuals, this would imply a change in the construct validity of the task when used between the motor and non-motor impaired.

Thus, the current study aimed to investigate, in a group of stroke patients with reduced upper limb dexterity, the relative extent to which cognitive and motor speed domains were being captured by three different general measures of speed with varying motor response requirements. Firstly, associations were explored between cognitive (indexed by perceptual/attentional speed on a non-motor task) and motor speed (indexed by dominant hand dexterity on a pegboard task), with performance on the three speed measures. Additional associations were also explored between neurological parameters with patients’ performances on all tasks. On the basis that speed measures have been shown to capture speed that is related to both cognitive and motor activities (11, 20), it is likely, at least in the control group, that significant correlations will be observed between both cognitive and motor speed with task performance. Secondly, the relative contribution of cognitive and motor speed indices toward the general speed measures was investigated using regression analyses. Similarly, based on the findings of previous regression analyses (11, 20), motor speed is likely to significantly contribute to the variance in the speed measures in the non-motor impaired (controls), and we hypothesize this contribution to be proportionally larger in the motor impaired (patients).

## Materials and Methods

### Participants

As part of a larger stroke project, 27 ischemic stroke (without neglect) and 10 Transient Ischemic Attack (TIA) patients were recruited from the stroke/TIA outpatient clinic at Footscray Hospital, Western Health, Australia. Project approval was obtained from Western Health Low Risk Human Research Ethics Committee (HREC/13/WH/105) and La Trobe University Human Research Ethics Committee. All participants provided written informed consent in accordance with the Declaration of Helsinki.

Inclusion criteria required that patients: (1) were aged between 40 and 80 years; (2) experienced an ischemic stroke or TIA; (3) had adequate ability to understand English, and therefore, the capacity to provide consent and understand task instructions; (4) had not previously been diagnosed with a neurological, psychiatric, or neurodevelopmental condition prior to the stroke/TIA; and (5) were not, at the time of recruitment, diagnosed with a psychiatric, or neurodegenerative condition.

This cohort of patients has previously been reported in Low et al. (22). However, assessments relevant to the current study were obtained 3 months post the initial recruitment and assessment as per Low et al. (22). The reason for a follow-up assessment was due to the fact that several stroke patients presented with severe hemiparesis during the initial session. Thus, motor-related tasks were not administered at the time.

Due to an attrition of 15% (3 months post-recruitment) and following exclusion of data from 2 left-handed patients, sample sizes were relatively smaller, with data from a total of 29 right-handed patients included for analysis. Nine patients had left hemisphere stroke (LHS) (*M* age = 53.44, SD = 9.30), 11 patients had right hemisphere stroke (RHS) (*M* age = 59.64, SD = 8.52), and 9 were TIA patients (*M* age = 61.89, SD = 6.75). Notably, patients who participated in this study were generally from a younger age range. Duration between hospital admission and time of the second assessment was an average of 329 days for the LHS group (SD = 122.98), 324 days for the RHS group (SD = 138.36) and 226 days for the TIA group (SD = 72.28). There was no significant difference in the time of the current assessment post-hospital admission between patient groups, *F* (2, 26) = 2.28, *p* = 0.12.

Thirty-one healthy individuals were recruited and seen on one occasion only. The same inclusion criteria were used for the recruitment of controls apart from items relating to the neurological event. This cohort of participants has similarly been reported in Low et al. (22). As per the patient group, data for 2 left-handed control participants were retrospectively excluded, leaving a total of 29 right-handed participants (*M* age = 54.72, SD = 8.89).

Demographical and clinical information, including NIHSS upon hospital presentation, scores on the 21-Item Depression Anxiety and Stress Scale (DASS-21) (23), and the Edinburgh Handedness Inventory (EHI) (24) are presented in Table 1. While not the most ideal method given the non-spherical nature of ischemic lesions (25, 26), a rough estimate of ischemic lesion volume was calculated from standard protocol imaging (CT and/or MRI) using the ABC/2 approach (27) and included in Table 1. More accurate lesion data were not available as not all patients within this setting routinely undergo CT perfusion or a diffusion-weighted imaging protocol. Note that NIHSS and lesion data were only available for stroke and not TIA patients. The DASS-21 and EHI were screening tasks administered during participant recruitment. See Low et al. (22) for more information regarding the screening tasks.

**Table 1:** Demographical and clinical information.

	Control (*n* = 29)	LHS (*n* = 9)	RHS (*n* = 11)	TIA (*n* = 9)
Age (years)	54.72 (8.89)	53.44 (9.30)	59.64 (8.52)	61.89 (6.75)
Education (years)	13.48 (2.17)	11.22 (2.64)	11.55 (3.05)	12.56 (4.13)
Gender (% male)	45	44	55	67
Days from insult	– (–)	329.00 (121.98)	323.64 (138.56)	226.22 (72.28)
NIHSS	– (–)	3.00 (2.92)	5.00 (5.69)	– (–)
Lesion volume (cm^3^)	– (–)	1.74 (2.58)	3.40 (8.99)	– (–)
DASS-21 Dep	1.42 (2.12)	3.78 (3.93)	1.78 (1.64)	2.89 (3.72)
DASS-21 Anx	1.15 (1.32)	2.78 (2.99)	1.56 (1.24)	3.44 (3.01)
DASS-21 Str	3.12 (2.86)	4.78 (3.46)	3.78 (2.05)	5.33 (4.80)
EHI	91.07 (14.43)	69.56 (43.56)	70.18 (38.59)	92.33 (8.56)
Pegboard R	13.26 (1.93)	11.67 (2.29)	11.33 (1.94)	11.67 (1.87)

*NIHSS, National Institutes of Health Stroke Scale; DASS-21, 21-item depression anxiety and stress scale; Dep, depressive symptom scale; Anx, anxiety symptom scale; Str, stress symptom scale; pegboard R, pegboard right-hand; LHS, left hemisphere stroke; RHS, right hemisphere stroke; TIA, transient ischemic attack; EHI, Edinburgh Handedness Inventory*.

*Dashes indicate where descriptive statistics were not applicable*.

Statistically, there was no significant difference in age between patient and control groups, *F* (3, 54) = 2.45, *p* = 0.07, η^2^ = 0.12, with a moderate to large effect size noted. There was no significant difference in education levels between patient and control groups, *F* (3, 54) = 2.25, *p* = 0.09, η^2^ = 0.11, with a moderate to large effect size noted. There was no significant difference in gender proportions in the LHS, RHS, and TIA groups compared with controls (45% male), χ^2^(1, *N* = 58) = 0.59, *p* = 0.44. On the DASS-21, no significant difference was found between patient and control groups for reported depressive symptoms (results based on Welch test given non-homogeneity of variance), *F* (3, 17) = 1.20, *p* = 0.34, anxiety symptoms (Welch test), *F* (3, 17) = 2.19, *p* = 0.13, nor stress symptoms (Welch test), *F* (3, 18) = 0.94, *p* = 0.44.

#### Documentation of Reduced Manual Dexterity in Patients

During the assessment, the Purdue Pegboard task (28) was administered to assess manual dexterity of the dominant right-hand in patients and controls. One-way ANOVA with planned contrast revealed a significant difference between groups, *F* (3, 54) = 3.90, *p* = 0.01, with mean right-hand pegboard score being significantly lower for all three LHS (*p* = 0.04, Cohen’s *d* = −0.80), RHS (*p* = 0.02, *d* = −0.97), and TIA groups (*p* = 0.04, *d* = −0.80) compared with controls (see Table 1). These results indicate that all three patient groups presented with significantly lower right-hand manual dexterity irrespective of the side and severity of the stroke. In this context, it was considered appropriate to further investigate the current research question by converging all patients into a clinical group with overall reduced right-hand dexterity. It should be noted that the current task was additionally used to provide an index of motor speed, and is described further in Section “Procedure and Materials.”

### Procedure and Materials

As per the approved protocol, the assessment occurred for approximately 1 hour in a quiet room and involved administration of a battery of tasks. Tasks were administered in the same order for each participant, and across both patient and control groups. The experimental design was an important aspect to consider and ensured that more important tasks were prioritized, especially since patients generally required longer times to complete tasks. Secondly, matching of task order across participants ensured that practice or fatigue effects, if present, occurred consistently across tasks.

Tasks relevant to the current study include a non-motor computerized speed measure to provide an index of cognitive speed, a task of manual dexterity (Purdue Pegboard task) to provide an index of motor speed, and three other general processing speed measures with varying levels of psychomotor demands. Processing speed measures were completed with the dominant right-hand. Measures are described below.

#### A Measure of Cognitive Speed

##### Inspection Time (IT)

The IT task is a psychophysical task that has been traditionally employed to measure perceptual speed (29). It utilizes an adaptive methodology that provides a threshold estimate, a value that indicates the minimal stimulus exposure duration required before reliable perception of the stimulus could be made (30). A core feature of this task is that performance does not require a psychomotor response component.

While the IT has been considered as a task measuring perceptual speed, recent studies have argued that the IT requires a degree of cognitive ability, including selective attention, to facilitate stimulus perception (31–33). This is in line with a body of evidence suggesting that the IT can be utilized as a biomarker of cognitive decline (34), given strong correlations between task performance with IQ and cognitive scores for short-term memory, working memory, and fluid reasoning (30, 35, 36). Therefore, given its non-motor paradigm, the dependent variable (i.e., stimulus exposure duration) of the task, at least in the current study, is used to provide an index of cognitive speed.

**Figure 1:**
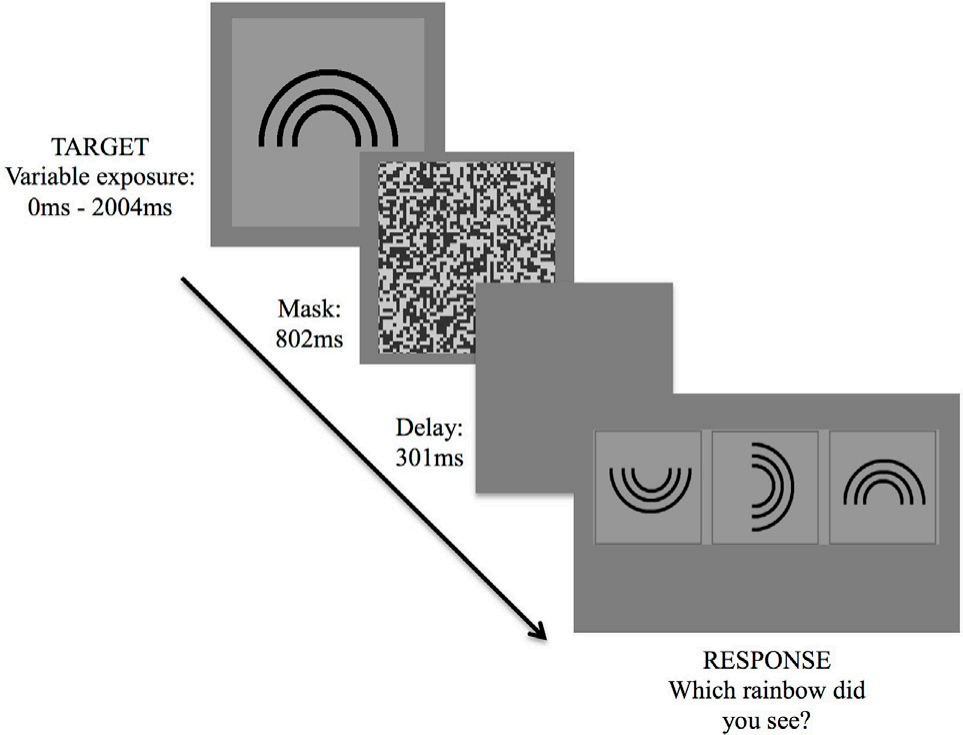
Inspection time task. Exposure of target stimulus followed by three forced-choice options, requiring participant to indicate the correct stimulus orientation.

The version of the IT task employed in this study was programmed *via* a specialized psychophysics program, VPixx (www.Vpixx.com), using a Parameter Estimation by Sequential Testing (PEST) technique. The task was presented on an Apple Mac laptop with a 15″ display monitor. Task stimuli were designed for a 57 cm viewing distance and consisted of a rainbow (target stimulus) of four different orientations (an upright standing rainbow, and rainbows rotated by 90, 180, and 270°, respectively), presented at random across trials (Figure 1). Before commencing, participants were seated comfortably, approximately 57 cm away from the laptop screen. Once participants were ready, the examiner provided some instructions and performed several trial attempts until participants understood task requirements.

Following commencement of the task, the target stimulus was presented for 2,004 ms, followed by a visual mask for 802 ms, a short delay of 301 ms, then a three forced-choice response option. Participants were asked to verbally indicate which of the three rainbows corresponded to the orientation of the rainbow that had been presented. The examiner then assisted by hitting a response key corresponding to the participant’s decision, which triggered the start of the next trial.

Threshold was determined by making use of the PEST routine. The paradigm employs a maximum-likelihood threshold estimation adaptive staircase algorithm similar to that described by Harvey (37), in which the dependent variable is adjusted down or up, depending on participants’ answers (i.e., whether correct or incorrect), changing the step size throughout the task. In short, on each trial, the actual threshold most likely to result in the sequence of all previous responses thus far is calculated. Exposure duration of the stimulus therefore fluctuated across trials until a threshold, in milliseconds, was reached. Termination of the routine was based upon the specified condition of having 90% confidence level that the threshold estimate fell within ±0.1 natural log units of the true threshold. Lower threshold levels imply shorter exposure times required to perceive the stimulus, thus a faster cognitive speed.

#### A Measure of Motor Speed

##### Purdue Pegboard

The Purdue Pegboard task provides a measure of manual dexterity and coordination of the upper limbs (28). The task comprises a wooden board with 2 centralized, vertical rows of 25 holes each, and pins that were placed in 2 cups, also centered at the top of the board. Participants were requested to place as many pins into the row of holes to the right of the board in 30 s, using their right-hand. This was repeated with the left-hand, then with both hands together (i.e., manual coordination). In the current study, right-hand dexterity score (total number of pins inserted using the right-hand within 30 s) was used to provide an index of dominant hand motor speed.

#### Measures of General Information Processing Speed Requiring Psychomotor Responses

##### Visuo-Motor Reaction Time (VMRT)

**Figure 2:**
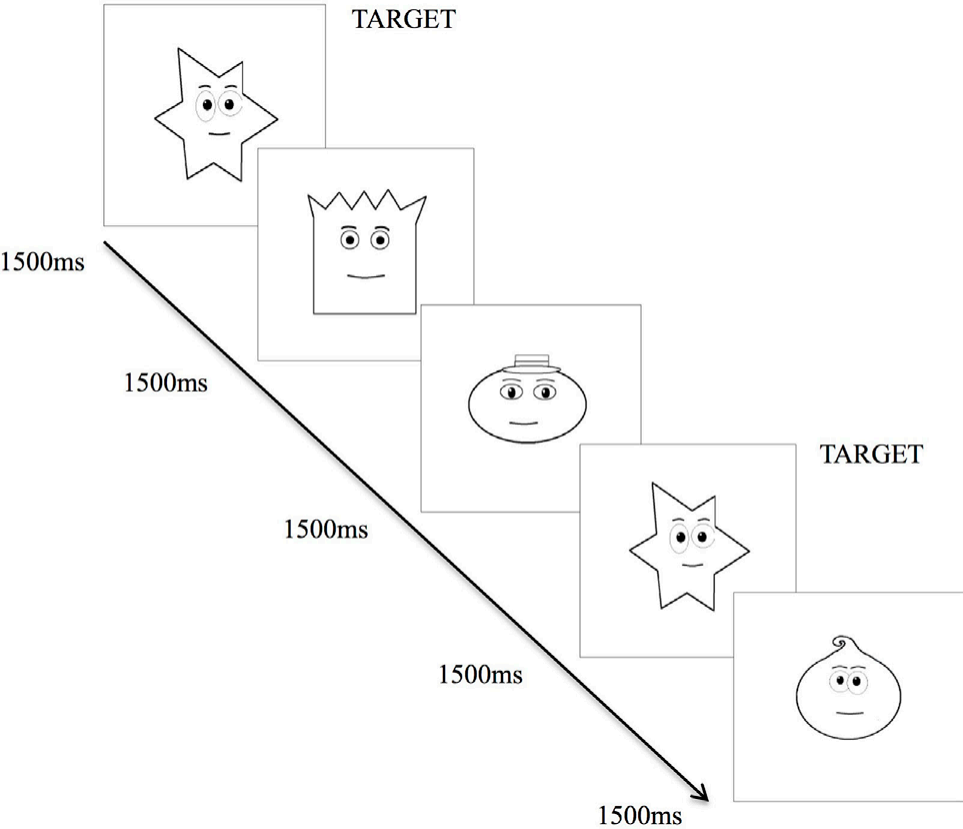
Visuo-motor reaction time task. Exposure of various cartooned faces requiring participant to hit the spacebar as soon as the target cartooned face appears.

The VMRT task provides a measure of general speed that is operationalized by reaction time of the dominant hand, in milliseconds, in responding to a stimulus following identification of the stimulus. The version of the VMRT task used in this study was similarly developed through the VPixx Program and employed an *n* = 0-back paradigm (38). Task consists of a series of 34 cartooned faces varying in shapes (i.e., round, square, star, and oval-shaped faces), presented centrally on the laptop screen (Figure 2). The faces subtended approximately 10 by 10° of visual angle at 57 cm from the laptop screen. In contrast to previously used versions (38, 39), the task was made easier for patients, whereby faces were presented sequentially at a longer duration of 1,500 ms per stimuli, with a 1,000 ms inter-stimulus interval.

Upon commencing the task, participants were required to tap the spacebar with their right-hand as quickly as possible, each time they saw the target (star-shaped) face. There were 34 trials corresponding to 34 faces, with 9 of the 34 faces being target faces. Reaction time for each participant was calculated by averaging the reaction time of each accurate target detection. Lower VMRT indicates a shorter reaction time to respond to the target stimulus, thus a faster speed of processing. This task has been described in another study (22).

Similar to the IT task, cognitive abilities including selective and sustained attention to the stimulus are integral for efficient task performance. However, response requirements of the VMRT meant that the outcome measure of the task would capture additional domains, including decision making time to arrive at the correct response selection and the time required for subsequent motor response execution. Given the use of an *n*-back paradigm, this task has therefore been previously and dually employed as a task of processing speed as well as a task measuring sustained attention and working memory (when *n* = 1 or *n* = 2 paradigms were incorporated) (40–42).

##### Symbol Search (SS)

The SS is a subtest from the WAIS-IV (14) that provides a measure of general speed of information processing. There are 60 items in total, each item comprising a set of 2 target symbols to the left of the page, and an array of 5 symbols and a “no” option box to the right of the page. Participants were requested to visually scan the target symbols, followed by the array of five symbols to determine if any of the five symbols matched either of the target symbols. Participants identified a matching symbol by crossing the symbol. If no matching symbols were present, participants were instructed to cross the “no” option. Participants were instructed to “work as fast as you can” for 2 min. The task was administered and scored in accordance with the WAIS-IV manual (14). Raw scores were used for analysis instead of scaled scores, with higher scores indicating faster speed of processing.

##### Coding (Cod)

The Coding is a subtest from the WAIS-IV (14) that provides a measure of general speed of information processing. With nine key codes listed at the top of the page indicating the digit–symbol pairs, participants were requested to inscribe symbols for each corresponding digit, ranging from one to nine. There were 135 digits in total. Participants were instructed to “work as fast as you can” for 2 min. The task was administered and scored in accordance with the WAIS-IV manual (14). Raw scores were used for analysis instead of scaled scores, with higher scores indicating faster speed of processing.

### Statistical Analysis

Pearson’s bivariate correlational analyses were performed to determine any significant associations between demographic variables (age and education levels), and cognitive and motor speed, with performance on the general speed measures (VMRT, SS and Cod) individually. Pearson’s bivariate correlational analyses were also performed between neurological parameters (NIHSS score and lesion volume) with performance on task, that is cognitive speed (IT), motor speed (Pegboard), and the speeded measures (VMRT, SS and Cod). Analyses were performed separately for the motor impaired clinical group and non-motor impaired control group.

To investigate the relative contribution of cognitive and motor speed on the general speed measures, hierarchical regression analyses were performed for clinical and control groups separately, on each measure, adjusting for age, and education. This statistical adjustment is considered to be important given the known influence of demographic factors on task performance (19, 43–45), as well as the rather large effect size observed here for group differences in age and education.

## Results

Means and standard deviations for performance on the cognitive, motor, and general speed measures are presented in Table 2. Patients required a longer stimulus exposure time on the IT compared with controls, indicating slower cognitive speed. Patients also presented with slower right-hand motor speed, which has been confirmed by planned contrast analysis demonstrating significantly lower right-hand dexterity in patients compared with controls. On the general speed measures, patients’ reaction times on the VMRT task were longer, and they obtained lower scores on the SS and Cod compared with controls, all of which indicate slower general speed of processing.

**Table 2:** Means, SDs, and performance ranges on tasks of interest.

	Clinical group (*n* = 29)			Control group (*n* = 29)		
	*M*	SD	Range	*M*	SD	Range
IT (ms)	23.17	17.25	7.75–71.90	18.61	12.66	3.61–47.65
Pegboard R	11.56	1.97	8.00–15.00	13.26	1.93	10.00–17.00
VMRT (ms)	652.32	180.04	432.60–1,167.50	543.17	67.94	406.60–710.90
SS	25.17	8.82	8.00–47.00	30.52	7.59	12.00–49.00
Cod	51.97	14.79	27.00–84.00	62.10	16.17	25.00–91.00

*IT, inspection time; pegboard R, pegboard right-hand; VMRT, visuo-motor reaction time; SS, symbol search; Cod, coding*.

### Correlational Analyses

#### Associations between Demographic Variables and Speed Indices with Speed Measures

**Figure 3:**
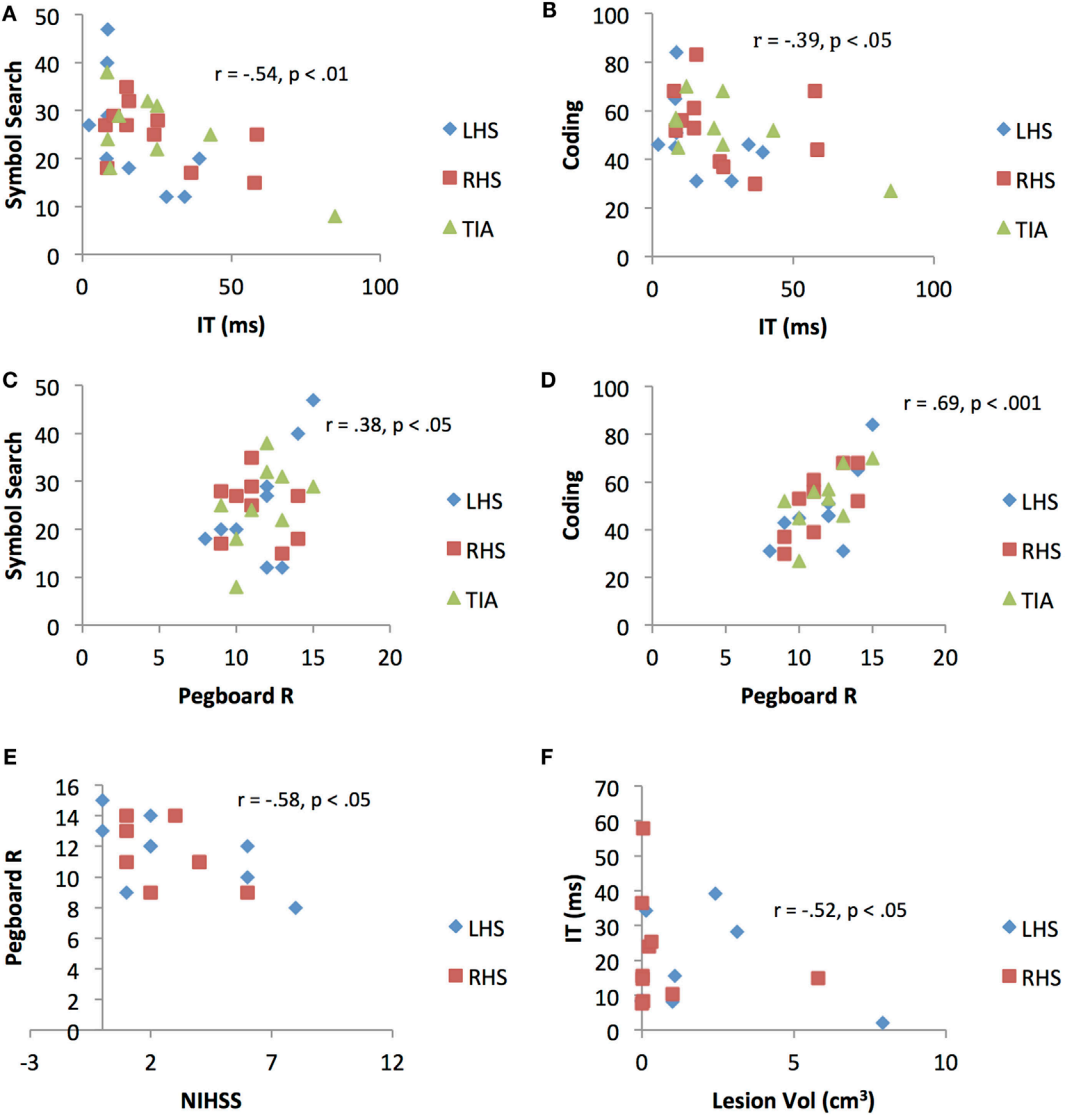
Scatterplots representing significant correlations between **(A)** Inspection Time (IT)/cognitive speed and Symbol Search (SS) performance **(B)** Inspection Time/cognitive speed and Coding performance **(C)** right-hand pegboard/motor dexterity and Symbol Search performance **(D)** right-hand Pegboard/motor dexterity and Coding performance **(E)** National Institutes of Health Stroke Scale score and right-hand Pegboard/motor dexterity performance, and **(F)** ischemic lesion volume and Inspection Time/cognitive speed performance. LHS, left hemisphere stroke; RHS, right hemisphere stroke; TIA, transient ischemic attack.

For the clinical group, significant associations were observed between all variables (age, education, cognitive speed, motor speed) with the paper-and-pencil tasks (SS and Cod), indicating that poorer task performance was associated with aging, lower levels of education, and slower cognitive (due to longer stimulus exposure durations) and motor speed. No significant correlations were observed for VMRT performance. For the control group, significant associations were observed between age, and cognitive and motor speed with the paper-and-pencil tasks. These results indicate that poorer task performance was associated with aging, and slower cognitive and motor speed. In contrast to the clinical group, no significant associations were observed between education levels with the paper-and-pencil tasks. For VMRT, no significant correlations were observed, with the exception of age being moderately and positively correlated with VMRT. Results are presented in Table 3. Significant results between cognitive and motor speed indices with the speed measures are presented in Figure 3.

**Table 3:** Correlations (*r*) between demographic variables and speed indices with general speed measures.

Variable	VMRT	SS	Cod
**Clinical group**			
Age	0.05	−0.53**	−0.36*
Education	−0.30	0.40*	0.33*
IT	0.07	−0.54**	−0.39*
Pegboard R	−0.25	0.38*	0.69***
**Control group**			
Age	0.32*	−0.47**	−0.38*
Education	−0.18	0.11	0.13
IT	0.14	−0.49**	−0.39*
Pegboard R	0.06	0.32*	0.33*

*VMRT, visuo-motor reaction time; SS, symbol search; Cod, coding; pegboard R, pegboard right-hand; IT, inspection time*.

*p < 0.05 (one-tailed).

**p < 0.01 (one-tailed).

***p < 0.001 (one-tailed).

#### Associations between Neurological Parameters with Speed Indices and Speed Measures (Clinical Group Only)

A significant association was observed between NIHSS score with motor speed (pegboard), *r* = −0.58, *p* < 0.05. A significant association was also observed between lesion volume with cognitive speed (IT), *r* = 0.52, *p* < 0.05. No associations were demonstrated between NIHSS score and lesion volume with the general measures (VMRT, SS and Cod). Results are presented in Figure 3.

### Hierarchical Multiple Regression Analyses

Given the moderate to large effect size when testing for differences in age and education levels between groups, and the known influence of age and education levels on speed outcomes (43, 44), hierarchical multiple regression analyses were conducted separately for clinical and control groups to explore the extent to which cognitive speed (IT) and motor speed (Pegboard R) individually predicts performance on the three measures (VMRT, SS and Cod), controlling for age and education.

Assumptions of linearity, normality, and homoscedasticity were assessed. Case wise diagnostics did not reveal any anomalous cases. Hierarchical regression results are described below, with *R* squared change, standardized beta and semi-partial (or part) correlations for each predictor variable presented in Table 4.

**Table 4:** Hierarchical regression of demographic variables and speed indices on general speed measures.

Dependent variable	Predictors		Clinical group			Control group		
			Δ*R*^2^	β	sr	Δ*R*^2^	β	sr
VMRT	Step 1	Age	0.09	−0.03	−0.03	0.15	0.35	0.34
		Education		−0.31	−0.29		−0.23	−0.23
	Step 2	IT	0.03	−0.10	−0.08	0.02	0.04	0.03
		Pegboard R		−0.17	−0.15		0.16	0.15

SS	Step 1	Age	0.35**	−0.46*	−0.44	0.25*	−0.49*	−0.49
		Education		0.28	0.27		0.18	0.17
	Step 2	IT	0.08	−0.32	−0.26	0.14	−0.34	−0.31
		Pegboard R		0.13	−0.11		0.19	0.17

Cod	Step 1	Age	0.19	−0.30	−0.29	0.18	−0.41*	−0.40
		Education		0.25	0.24		0.19	0.19
	Step 2	IT	0.34**	−0.20	−0.16	0.12	−0.26	−0.23
		Pegboard R		0.63**	0.55		0.24	0.22

Dependent variables are listed in order of increasing motor execution requirements.

VMRT, visuo-motor reaction time; SS, symbol search; Cod, coding; pegboard R, pegboard right-hand; IT, inspection time.

*p < 0.05.

**p < 0.01.

#### Visuo-Motor Reaction Time

For the clinical group, age and education levels at Step 1 explained 8.9% of the variance in mean VMRT, *F* (2, 21) = 1.03, *p* = 0.37. The entry of mean IT threshold and mean right-hand pegboard score at Step 2 explained an additional, but not statistically significant 2.9% of the variance in mean VMRT, after controlling for age and education levels, *F change* (2, 19) = 0.31, *p* = 0.73. For the control group, age and education levels at Step 1 explained 15.0% of the variance in mean VMRT, *F* (2, 24) = 2.12, *p* = 0.14. The entry of mean IT threshold and mean right-hand pegboard score at Step 2 explained an additional, but not statistically significant 2.2% of the variance in mean VMRT, after controlling for age and education levels, *F change* (2, 22) = 0.29, *p* = 0.75.

#### Symbol Search

For the clinical group, age and education levels at Step 1 explained a statistically significant 35.4% of the variance in mean SS score, *F* (2, 24) = 6.58, *p* = 0.01, with age being a statistically significant variable. The entry of mean IT threshold and mean right-hand pegboard score at Step 2 explained an additional, but not statistically significant 8.3% of the variance in mean SS score, after controlling for age and education levels, *F change* (2, 22) = 1.63, *p* = 0.22. For the control group, age and education levels at Step 1 explained a statistically significant 25.0% of the variance in mean SS score, *F* (2, 24) = 3.99, *p* = 0.03, with age being a statistically significant variable. The entry of mean IT threshold and mean right-hand pegboard score at Step 2 explained an additional, but not statistically significant 13.6% of the variance in mean SS score, after controlling for age and education levels, *F change* (2, 22) = 2.43, *p* = 0.11.

#### Coding

For the clinical group, age and education levels at Step 1 explained 19.1% of the variance in mean Cod score, *F* (2, 24) = 2.83, *p* = 0.08. The entry of mean IT threshold and mean right-hand pegboard score at Step 2 explained an additional statistically significant 33.6% of the variance in mean Cod score, after controlling for age and education levels, *F change* (2, 22) = 7.79, *p* = 0.003. In this final model, mean right-hand pegboard score was a statistically significant variable. For the control group, age and education levels at Step 1 explained 18.0% of the variance in mean Cod score, *F* (2, 24) = 2.64, *p* = 0.09. Age was found to be a statistically significant variable. The entry of mean IT threshold and mean right-hand pegboard score at Step 2 explained an additional, but not statistically significant 11.4% of the variance in mean Cod score, after controlling for age and education levels, *F change* (2, 22) = 1.77, *p* = 0.19.

## Discussion

With many currently available and commonly used information processing speed measures, the extent to which performance on task is explained more largely by motor output speed rather than cognitive speed, especially in individuals with upper limb deficits, is unclear. In the current study, a total of five tasks were administered to the clinical group (between 7 and 11 months post-stroke/TIA) and to their neurologically healthy counterpart, two of which were to provide an index of cognitive and motor speed, respectively, and the remaining three were the processing speed measures (requiring psychomotor responses) being examined.

Correlations were first performed between cognitive speed (IT) and dominant right-hand motor speed (Pegboard R) with performance on the processing speed measures (VMRT, SS, and Cod). In line with our hypothesis and previous findings, significant associations were demonstrated between both speed indices with the tasks (though only the paper-and-pencil measures), and for both patients and controls (11, 20). Further, the observed correlations between cognitive and motor speed with Symbol Search and Coding were marginally stronger in the motor impaired clinical group compared with controls. This was especially the case for right-hand motor speed and Coding performance in the clinical group (Table 3), implying that compromised hand dexterity does accentuate the strength of the association. In line with this finding is the fact that recent imaging studies have also shown the need to recruit extra contralesional neural resources to facilitate motor functioning, even after a year post the initial ischemic injury (46–50). Thus, it is likely that motor execution abilities do play a vital role in influencing the rate of task performance outcomes. Similarly, it is also likely that residual cognitive abilities are poorer in patients compared with controls (51–53), thus contributing to the marginally stronger associations between slower cognitive speed with poorer performance (operationalized by lower raw scores) on Symbol Search and Coding.

More interestingly, further correlational analyses between neurological parameters with task performance revealed a significant correlation between (1) neurological symptom severity immediately post-stroke onset with right-hand motor speed and (2) a gross estimate of lesion volume with cognitive speed. The distinction between these associations is one to be emphasized, as they provide novel insights into the likelihood of a heavier motor (or motor speed) loading on NIHSS measurement, while cognitive speed in itself is more likely to be intrinsically linked to injury severity by way of the extent of the ischemic lesion.

In this study, hierarchical regression analyses were performed to determine the relative extent to which the variance in general speed measures can be significantly explained by both cognitive and motor speed indices. Previous studies have found that, within the general population, motor speed as a single construct significantly predicted performance on a range of paper-and-pencil speed measures, including WAIS-III Coding and Symbol Search subtests, Symbol Digit Modalities Test, and the Trail Making Test (11, 20). Surprisingly, this finding was not replicated with our control participants, with a likely reason being insufficient statistical power resulting in false negatives. In fact, this argument was supported by supplementary analyses performed with a larger sample size (*via* pooling of both patient and control participants), whereby a significant effect of motor speed on Coding was demonstrated (β = 0.434, *p* = 0.001).

It is important to note that, while right-hand motor speed did not significantly predict Coding performance in the control group, the opposite was demonstrated for the patient group, with the variance in Coding outcomes being significantly and largely explained by motor speed. Thus, in the presence of motor difficulties, Coding as a general processing speed task may increasingly capture the motor speed deficits more so than cognitive speed. In this context, it may be the case that the task’s construct validity gradually changes with increasing upper limb motor difficulties, especially when the task involves a high motor response load. This should be a matter of clinical concern since such tasks become of minimal value in informing residual cognitive speed following an ischemic insult. Yet, paradoxically, the Coding task (and Symbol Search also) is used by clinicians on an everyday basis to assess processing speed deficits from a cognitive perspective.

Another pattern of results worth discussing relates to the VMRT task. Specifically, VMRT performance was neither associated with cognitive nor motor speed, and was also not significantly predicted by either of these speed domains. The non-contribution of cognitive speed toward VMRT is not particularly unexpected, given that the task paradigm did not involve any manipulation or thresholding of the stimulus exposure time (as with the IT task) to enable precise indication of speed that is related to a cognitive component. Furthermore, as mentioned earlier, the VMRT task used in the current study is very much analogous to a 0-back task, the latter known to be better at indicating one’s ability to sustain attention over time (40, 41). The non-contribution of motor speed toward VMRT, on the contrary, could very well reflect minimal motor requirements of the task, relative to Symbol Search and Coding. This explanation is consistent, and can be corroborated by the incremental increase in standardized beta weights as a function of increased motor demands across tasks (e.g., β of 0.16 for VMRT, 0.19 for SS, and 0.24 for Cod, for the control group).

Overall, it should be emphasized that the contribution of motor speed components on processing speed task performance has already been established in healthy individuals (11, 17, 20, 21), with which the current findings serve to indicate similar but stronger links in stroke patients. Our findings pose significant implications both clinically and in research, and may imply a degree of misinterpretation of results where such tasks have been used to measure cognitive speed. For example, the broader stroke literature has often described attention and processing speed as being most commonly and severely affected post-stroke (54–56), without much account for the form of speeded task that was used. In a study investigating the rate of cognitive decline post-stroke, and across carriers and non-carriers of apolipoprotein E ε4 (APoE ε4), findings indicate an association between the ApoE with declines in processing speed, the caveat being that speed was measured by the Coding task (57). Finally, another study by Wagle et al. (58) revealed that Coding raw scores at baseline explained 42% of the variance in modified Rankin Scale (mRS) scores. However, these findings may have in fact implied a link between motor efficiency with mRS outcomes.

On the basis of the above arguments, we propose that the use of a non-motor threshold paradigm (as with the IT task) may potentially serve as a better indicator of cognitive speed. Our argument may be supported by a few key findings. Firstly, IT performance was significantly associated with that of Symbol Search and Coding, implying a level of consistency in the underling cognitive constructs that are being captured by both the IT and the paper-and-pencil tasks. Secondly, IT was also found to be significantly associated with injury severity based on grossly estimated lesion volume. In fact, the utility of the IT task in measuring cognitive speed has been studied in intracranial tumor patients and in individuals with autism spectrum disorders, with favorable outcomes observed (59, 60). Therefore, we argue for the need to further investigate the utility of this task in a stroke sample, as it may have clinical value in measuring cognitive-related speed, especially in the motor impaired.

One limitation to the current study is that sample sizes were smaller, and the numbers were further reduced by attrition and elimination of left-handed participants. Therefore, whether additional significant predicting effects may be observed in a larger sample with upper limb difficulties will require further investigation. It is also worthwhile mentioning, to our surprise, that all patient sub-groups (i.e., LHS, RHS and TIA) individually presented with reduced dominant hand dexterity regardless of the side and severity of the stroke. We hypothesize that this is possibly related to a range of pre-existing and post-stroke factors including small vessel ischemic changes, post-stroke psychological sequelae, and fatigue, and perhaps also inter-hemispheric disruption and subsequent cortical reorganization. In contrast to previous understandings of the neurobiological principles of motor recovery (47), for example, more recent evidence appears to indicate a favorable relationship between increased contralesional motor cortex activity with recovery of the affected upper limb (46). Thus, while increased contralesional activation may facilitate recovery of the contralesional limb, its concurrent consequence is a disruption to the default inter-hemispheric activity that may concurrently impact on motor function of the ipsilesional limb. Future studies again, will benefit from further examining the contribution of these factors toward motor dexterity.

In conclusion, a number of key findings must be reiterated. Firstly, significant associations between cognitive and motor speed indices with paper-and-pencil measures were demonstrated irrespective of motor compromise or otherwise (i.e., in both clinical and control groups), indicating that both speed indices are intrinsically associated with the cognitive and motor components of the tasks. Secondly, findings from our regression analyses served to validate the notion that processing speed outcomes on the Coding task were predominantly measuring motor-related speed deficits in the presence of motor difficulties of the dominant hand. Thus, the overall findings highlight the importance of employing measures of speed that are not confounded by any motor requirements, especially when used in individuals with motor difficulties. We would like to emphasize that the use of more appropriate speed measures in clinical settings is particularly important when characterization of speed is aimed at painting a cognitive picture rather than one that is based on motor and physical abilities.

## Ethics Statement

Project approval was obtained from Western Health Low Risk Human Research Ethics Committee (HREC/13/WH/105) and La Trobe University Human Research Ethics Committee. All participants provided written informed consent in accordance with the Declaration of Helsinki.

## Author Contributions

EL designed and executed the study, analyzed the data, and prepared the manuscript. SC designed, supervised and managed the study, and made important theoretical contributions. BO supervised the analysis of data and made important theoretical contributions. DP faciliated study logistics, supervised the study on site and made important theoretical contributions. TW facilitated study logistics, supervised the study on site, and made important theoretical contributions. All authors were involved in the proofreading and revision of this manuscript.

## Conflict of Interest Statement

The authors declare that the study was conducted in the absence of any commercial or financial relationships that could be construed as a potential conflict of interest.
